# A Randomized, Double Blind, Placebo Controlled, Parallel-Group Study to Evaluate the Safety and Efficacy of Curene® versus Placebo in Reducing Symptoms of Knee OA

**DOI:** 10.1155/2018/5291945

**Published:** 2018-10-25

**Authors:** Sanjib kumar Panda, Somashekara Nirvanashetty, Vivek A. Parachur, Nilima Mohanty, Tathastu Swain

**Affiliations:** ^1^Head Operation, Ocius Life Sciences Pvt. Ltd., Chennai 600029, India; ^2^CSO, Olene Life Sciences Pvt. Ltd., Chennai 600058, India; ^3^CEO, Olene Life Sciences Pvt. Ltd., 600058, India; ^4^Associate Scientist, Olene Life Sciences Pvt. Ltd., 600058, India; ^5^Senior CRA, Ocius Life Sciences Pvt. Ltd., 600029, India

## Abstract

**Background:**

Curene® is a bioavailable formulation of turmeric * Curcucma longa* extract comprising naturally derived curcuminoids formulated with proprietary Aquasome® technology. Curcuminoids were found to have anti-inflammatory properties by inhibiting Cyclooxygenase-2 (COX-2) and 5-lipoxygenase (5-LOX) enzyme and hence have potential application in the treatment of Osteoarthritis (OA). To evaluate the safety and efficacy of Curene® a randomized, double blind, placebo controlled, parallel-group study was conducted in subjects with knee OA. Significant improvements in clinical endpoints were observed during the trial along with excellent safety profile.

**Methods:**

Fifty (50) subjects aged between 40 and 75 years who were suffering from unilateral or bilateral OA of the knee for greater than 3 months according to American College of Rheumatology (ACR) criteria were enrolled. They were randomized into two treatment groups; one group received Curene® 500 mg once daily and the other group received placebo. Efficacy was evaluated using change from baseline in Visual Analogue Scale (VAS) and Western Ontario and McMaster Universities Osteoarthritis Index (WOMAC) score. Biochemical and hematological parameters including urine analysis were performed to evaluate the safety of Curene® in OA patients.

**Result:**

Forty-six (46) subjects completed the study. The reduction from baseline in total WOMAC score (also subscale scores) and VAS score resulted in statistically significant difference when compared to placebo. It was also found to be safe and well tolerated as there was no incidence of treatment related AEs.

**Conclusion:**

Curene® results in statistically significant and clinically meaningful reduction in pain, stiffness, and improvement in physical functioning in subjects suffering from knee OA. Curene® also demonstrates excellent safety profile during the study.

**Trial Registration:**

This trial is registered with Clinical Trial Registry, India, CTRI/2017/07/009044, registered on 14th July 2017, http://ctri.nic.in/Clinicaltrials/showallp.php?mid1=19264&EncHid=&userName=ocius%20life%20sciences.

## 1. Introduction

Osteoarthritis is a common, chronic, progressive, skeletal, and degenerative disorder that commonly affects the knee joint which causes the disability of elderly people in developed countries [1].* Curcuma longa*, the rhizome from which the curcuminoids are extracted and purified, has been traditionally used as an anti-inflammatory agent in many Ayurvedic medicines for several years [2]. Curcuminoids possess significant anti-inflammatory activity by interacting with many inflammatory processes. Curcumin inhibits inflammatory cytokines, such as interleukins (ILs) and chemokines, as well as inflammatory enzymes such as cycloxygenase-2 (COX-2), inducible nitric oxide synthase (iNOS), and other molecules as cyclinD1. This natural anti-inflammatory agent is able to inhibit Microtubule Associated Protein Kinase (MAPK) and Nuclear Factor kappa-light-chain-enhancer of activated B cells (NF-*κ*B) pathways in Tumor Necrosis Factor (TNF*α*) treated HaCaT cells and, therefore, IL-1*β* and IL-6 expression. In others studies in BV2 microglia cell stimulated with lipopolysaccharide (LPS), curcumin also inhibited IL6 and TNF-*α* [3]. Apart from that it has antioxidant property as it facilitates scavenging different forms of free radicals, such as reactive oxygen and nitrogen species. It can also modulate the activity of glutathione (GSH), catalase, and superoxide dismutase (SOD) enzymes active in the neutralization of free radicals [4]. Free radicals are produced in diseased and infectious state of body which disturb the body's metabolism. Curcumin is a potent inhibitor of the production of inflammatory and catabolic mediators by chondrocytes. In addition, it also inhibits the macrophage migration inhibitory factor (MIF) induced upregulation of matrix metalloproteinases MMP-1 (interstitial collagenase) and MMP-3 (stromelysin), which plays a significant role in the damage caused to the cartilage [5], signifying that this natural compound could be efficient in the treatment of OA.

OA is the single most common cause of disability in older adults. An estimated 10% to 15% of all adults aged over 60 have some degree of OA, with prevalence higher among women than men. According to the United Nations, by 2050 people aged over 60 will account for more than 20% of world's population. Out of 20% a conservative estimate of 15% will have symptomatic OA and one-third of these people will be severely disabled. This means that by 2050, 130 million people will suffer from OA worldwide, of whom 40 million will be severely disabled by the disease [6]. Knee OA is one of the main causes of devastated mobility in elderly people. Since there is no cure for OA, treatment focuses on reducing symptoms especially pain [7].

The existing pharmaceutical option for OA includes NSAIDs that is to be used for a long-term basis. Using NSAIDs for a long term is well-known for their adverse drug reaction. Approximately 30% of hospitalizations occur due to adverse drug reactions that are caused by NSAIDs. Major adverse effects include but are not limited to GI bleeding, hypertension, congestive heart failure, hyperkalemia, and renal insufficiency [8, 9]. Although some of these disadvantages can be avoided by using paracetamol or selective COX-2 inhibitors yet, long-term use of paracetamol and COX-2 inhibitors could lead to hepatotoxicity, chronic renal impairment, and cardiovascular side effects.

Curene® has been successfully developed by using a novel technology that overcomes the side effects/drawbacks associated with NSAIDs which allows the natural active ingredient to cross the cell membrane to impart its therapeutic benefits efficiently.

Several OA trials have been conducted to evaluate the safety and efficacy of curcumin based formulations in alleviating the symptoms of pain and inflammation associated with OA. Many of these trials have been carried out using patented bioavailable curcuminoids formulations like Meriva® [10], BCM-95® [11], Curcumin C3 Complex®, etc. The results of these studies have indicated that the natural approach was superior over prescription drugs on pain parameters and if given on top of prescription medicine, it reduced the symptoms of knee OA. Decreased joint pain and improvement in joint function were observed in OA patients involved in the studies. Significant improvement of clinical and biochemical endpoints was also observed with the use of bioavailable curcumin. The proposed study was to validate the clinical data for the efficacy and safety of Curene® in subject with OA of Knee.

Curene® is a unique formulation of turmeric* Curcuma longa* extract comprising curcuminoids. It is developed using proprietary technology called Aqueosome® technology for enhancing the bioavailability of curcuminoids. It belongs to a group of flavonoids which are known to exhibit significant anti-inflammatory and anti-oxidant activity with inhibitory effect on many cytokines like TNF-*α*, IL-1*β*, IL-6 [12], etc. Several* in vivo* studies have demonstrated a marked inhibition of inflammatory mediators in animals treated with curcumin and thereby suggesting its role in reducing the inflammatory symptoms associated with OA.

## 2. Materials and Methods

### 2.1. Recruitment of Patients

**Figure 1:**
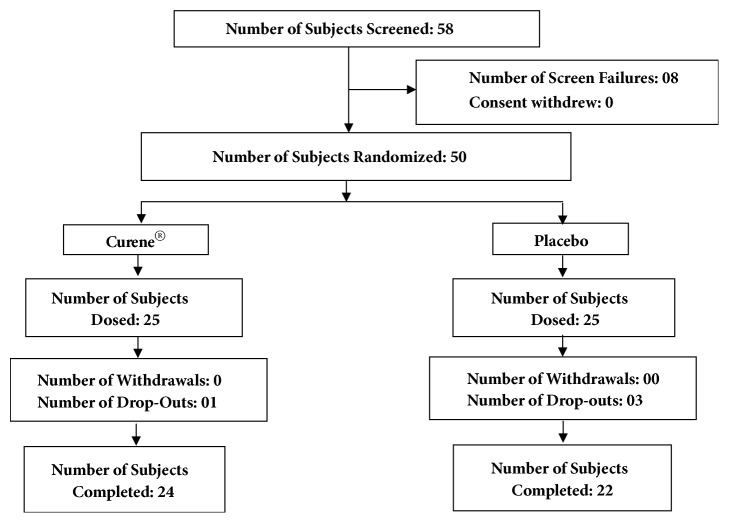
Flow chart of patients participating in study.

This clinical trial was performed at Vijaya Super Specialty Hospital, Andhra Pradesh, India, from June 2017 to September 2017 (clinical trial registration number: CTRI/2017/07/009044). The study protocol was evaluated and approved by the Vijaya Ethics Committee. An overview of the clinical study is provided in Figure 1. Fifty patients aged 40-75 years who were suffering from unilateral or bilateral OA of the knee for greater than 3 months (ACR criteria) were enrolled after meeting the inclusion and exclusion criteria (described in Table 1). All patients signed the Institutional Review Board approved consent form. After selection, the patients were randomly distributed into two groups; patients' demographic data are summarized in Table 2. At screening visit patients were evaluated for disease severity using VAS score and WOMAC scores. Double-blinded study was to avoid the bias and it helps to prevent any psychological effect.

### 2.2. Study Design

A randomized, double blind, placebo controlled, parallel-group study was conducted to evaluate the safety and efficacy of Curene® versus placebo in reducing symptoms of knee OA. Each subject was randomly assigned to a treatment group using randomization table generated using validated computer software. The subjects were allocated to two different arms: placebo (n=25); Curene® (n=25), in which subjects received one capsule of 500 mg once daily. Subjects in the placebo group received one capsule that resembles the active group filled with Micro Crystalline Cellulose. Each subject acknowledged a questionnaire providing details regarding their symptoms of pain, stiffness, and physical function at day -7 to 0 (screening visit), day 1 (randomization visit), day 7 (visit 2), day 14 (visit 3), day 30 (visit 4), and day 60 (visit 5), respectively.

### 2.3. Efficacy Assessment

#### 2.3.1. Primary Efficacy Assessment

Efficacy was based primarily on the mean change from baseline to end of treatment in the total WOMAC score. It was assessed in screening, randomization and in every follow-up visit. If any participants had OA in both knees, they were instructed to choose the more painful knee as the primary knee. The higher the scores, the greater the pain and stiffness and the worse the physical function. Intention to treat (ITT) populations was used for the primary efficacy analysis.

#### 2.3.2. Secondary Efficacy Assessment

Secondary endpoints were changed from baseline in VAS score and WOMAC subscale score (pain, stiffness, and physical function). Both parameters were assessed in screening and every follow-up visit. VAS used here was a horizontal line marked from 0 to 100 mm, anchored by word descriptors at each end like 0 to 4 mm can be considered no pain; 5 to 44 mm, mild pain; 45 to 74 mm, moderate pain; and 75 to 100 mm, severe pain. Subjects were asked to mark on this line in accordance with relevant amount of pain they were experiencing and the value was noted by the investigator in mm.

The individual components of the WOMAC subscale include pain, stiffness, and physical function and were assessed for secondary efficacy parameter. Treatment effectiveness was assessed by both investigator and subject using a series of questionnaire that incorporated the following elements: pain (5 questions, during walking, using stairs, in bed, sitting, and standing), stiffness (2 questions, during wake up, resting later in the day), and physical function (17 questions). ITT populations were used for the evaluation of secondary efficacy analysis.

### 2.4. Safety Assessment

Safety assessment included reporting of AEs, clinical laboratory assessment, vital signs, and physical examination. The entire population under treatment was included in the safety analysis. A complete medical history was taken during the screening period and updated throughout the study period at each visit.

### 2.5. Rescue Medication

Paracetamol (2000mg/day) was used as rescue medication based on the investigator's advice. Use of rescue medication was checked and recorded in the source documents, subject daily diary card, and case report form in each visit. Subjects were not allowed to take rescue medication 24 hours before the site visit.

### 2.6. Statistical Analyses

Detail statistical analyses were performed by using SAS software to evaluate the efficacy of Curene® in comparison with the placebo group in terms of improvement in pain, stiffness, and physical function scores at baseline, day 7, day 15, day 30, and day 60 of treatment. Pair-wise changes were examined by carrying out a least significant difference test for all possible pairs. The significance of the effects of the treatment groups was compared by using one-way analysis of covariance (ANCOVA). Results with P < 0.05 are considered statistically significant.

## 3. Result

### 3.1. Demographic and Other Baseline Characteristics

Descriptive statistics comparing demographic variables and baseline outcome measures (that includes VAS, total WOMAC, and WOMAC subscale scores) are described in Table 3. There were no significant differences at baseline between both groups regarding age, height, weight, body mass index (BMI), VAS, total WOMAC, and WOMAC subscale score.

### 3.2. Clinical Efficacy

The scores between the Curene® and placebo treated groups were obtained and compared throughout the study period. The active treated group ensured clinically and statistically significant improvement in pain, stiffness, and physical function scores in OA subjects at days 7, 15, 30, and 60 when compared with baseline.

Curene® exhibited statistically significant improvement in all parameters as early as 7 days after the start of treatment in comparison to placebo group and this was maintained through the entire study period. It showed highly statistical significant (p<0.05) improvements by 51.32%, 47.94%, 48.06%, 53.1%, and 52.07% in VAS, total WOMAC, and WOMAC subscale scores (pain, stiffness, and physical function), respectively.

#### 3.2.1. Primary Efficacy Analyses

Curene® treated group was superior over placebo in percentage reduction in WOMAC total score from baseline which was found to be 7.92% at visit 2, 17% at visit 3, 31.78% at visit 4, and 51.32% at visit 5 (Table 4).

#### 3.2.2. Secondary Efficacy Analyses

Both VAS and WOMAC subscale scores decreased progressively from baseline to end of the study in Curene® group when compared with placebo. Percentage reduction in VAS score was found to be statistically and clinically significant, i.e., 11.03% at visit 2, 20.3% at visit 3, 30.06% at visit 4, and 47.94% at visit 5 in Curene® treated group (Table 5).

In Curene® treatment group the percentage reduction in WOMAC pain was 10.68%, 19.9%, 29.61%, and 48.06% at visit 2, visit 3, visit 4, and visit 5, respectively (Table 6). Likewise, the percentage reduction in stiffness was 7.97% at visit 2, 22.12% at visit 3, 37.17% at visit 4, and 53.1% at visit 5 (Table 7). The percentage reduction in WOMAC physical function score was found to be 7.01% at visit 2, 15.13% at visit 3, 31.53% at visit 4, and 52.07% at visit 5 (Table 8).

### 3.3. Rescue Medication Usage

Overall 7 (28%) subjects used rescue medication in the placebo treatment group, whereas this number was found to be 3 (12%) in Curene® treated group. The total number of rescue medications taken in Curene® treated group was only 19 tablets of 500 mg paracetamol, whereas in the placebo treated group it was 75 tablets of 500 mg paracetamol. There was a significantly minimal use of rescue medication in the Curene® group compared to the placebo group.

### 3.4. Safety Assessments

The overall incidence of AEs was 12% (3/25 subjects) and 8% (2/25 subjects) in the Curene® and placebo group, respectively. There was no statistically significant difference in the incidence of AEs between the Curene® and placebo group. All the AEs were mild in severity in both treatment groups and no clinically relevant AEs were identified. The AEs in Curene® group were abdominal pain 1 (4%), bloating 1 (4%), and headache 1 (4%) whereas GERD 1 (4%) and headache 1 (4%) were observed in the placebo group. Though there were some AEs found in the active group and in the placebo group, all the subjects were found to be in stable condition. There were no notable changes in urine analysis parameters, vital signs, physical examination, and systemic examination (Table 9).

### 3.5. Dropout

Only one subject from Curene® treated and 3 subjects from placebo treated group were excluded from the study as they were lost to follow-up.

## 4. Discussion

In the modern age of medicine, various kinds of anti-inflammatory and analgesic are available. These medicines have their adverse effects which are associated with significant morbidity and mortality in the older population and hence cannot be used for a longer period of time as a treatment of choice. Most of the clinical trials conducted so far with curcuminoids based formulations for the management of OA are done with 1 gm or more than 1 gm per day dosing but Curene® is developed to initiate its action with 500 mg per day with a novel mechanism to provide sustained release of its active curcumin and hence the study was planned to evaluate efficacy of 500 mg once daily dose of Curene® for the management of OA in the current clinical study.

The study demonstrated that Curene® has potential efficacy in terms of reducing pain, stiffness, and improving physical function of OA subjects. In addition to check improvements in the treatment groups data for all parameters were compared between baseline and every follow-up visits.

Efficacy evaluation of the Curene® demonstrated superiority over the placebo treatment in all of the clinical assessment tests performed for the WOMAC as well as the VAS. Evaluation of the clinical endpoints, such as the total WOMAC, WOMAC physical functioning, WOMAC pain, WOMAC stiffness, and VAS, demonstrated statistical significance (p< 0.05) improvement of the Curene® group over the placebo group. Paired t-test revealed that treatment group has highly statistically significant improvements in all parameters over placebo. Based on the result of this study it was concluded that Curene® can be effective in reducing symptoms of OA and also it is safe and well tolerated. No clinically relevant AEs were identified in subjects with OA.

## 5. Conclusions

In summary, the present study validates the efficacy and safety of Curene® in the management of symptoms of OA. The present study also confirms the quick onset of therapeutic action of Curene® in OA subjects along with significant improvements in clinical end points compared to placebo. It significantly reduces the joint pain and improves the joint function as early as 7 days of treatment. This study validates a potential application of Curene® as a useful alternative treatment option for the management of symptoms of OA.

## Figures and Tables

**Table 1:** Inclusion and exclusion criteria.

Criteria	Details

Inclusion	(i) Male and female subjects 40 – 75 years of age with a Body Mass Index (BMI) of approximately 18 to 30 kg/m2.
	(ii) Unilateral or bilateral OA of the knee for greater than 3 months (ACR criteria)
	(iii) Subjects with radio graphic evidence by Kellgren - Lawrence grade 2 or 3
	(iv) Female subjects of childbearing potential must be using a medically acceptable form of birth control. Female subjects of non-childbearing potential must be amenorrheic for at least 1 year or had a hysterectomy and/or bilateral oophorectomy
	(v) VAS score during the most painful knee movement between 40-70 mm
	(vi) Subjects having mild-to-moderate pain not adequately or completely controlled with anti-inflammatory drugs
	(vii) Results of screening are within normal range or considered not clinically significant by the Principal Investigator
	(viii) Drug naive subjects or subjects willing to refrain from using ibuprofen, aspirin or other NSAIDS (other than acetaminophen/paracetamol as rescue) or any other pain reliever including topical application (OTC or prescription) and Omega 3 fatty acids during the entire trial.
	(ix) Willing to sign the informed consent and comply with study procedure

Exclusion	(i) Female subjects, who are pregnant, breast feeding or planning to become pregnant.
	(ii) Subject has known allergy to non-steroidal anti-inflammatory drugs (NSAIDs) (including aspirin) or has a suspected hypersensitivity, allergy or other contraindication to any compound present in the study medication
	(iii) History of underlying inflammatory arthropathy or severe RA or OA
	(iv) Subjects scheduled for any surgery within 3 months of completing the study
	(v) Recent injury in the area affected by OA of the knee (past 4 months)
	(vi) History of Gout
	(vii) History of congestive heart failure
	(viii) Evidence or history of clinically significant (in the judgment of the Investigator) hematological, renal, endocrine, pulmonary, gastrointestinal, cardiovascular, hepatic, neurologic diseases, or malignancies
	(ix) History of Systemic Lupus Erythematosus (SLE)
	(x) High alcohol intake (>2 standard drinks per day) or use of recreational drugs (such as cocaine, methamphetamine, marijuana, etc.)
	(xi) History of psychiatric disorder that may impair the ability of subjects to provide written informed consent
	(xii) Participation in any other trials involving investigational or marketed products within 30days prior to the Screening Visit
	(xiii) Have taken any corticosteroid, indomethacin, Glucosamine + chondroitin, within 3 months prior to the Treatment Period, Day 0 (Visit 1) or intra-articular treatment / injections with corticosteroid or hyaluronic acid or Omega-3 Fatty acids dietary supplements within 6 months preceding the treatment period.

**Table 2:** Demographic characteristics.

Demographic Parameters	Curene®	Placebo
	(N=25)	(N=25)
Age (years)	55.20±8.58	53.12±8.25
Height (cm)	164.96±8.31	167.16±6.02
Weight (Kg)	68.96±6.46	69.56±5.68
BMI (Kg/m^2^)	25.44±2.75	24.92±1.92

**Table 3:** Baseline characteristics.

Efficacy Parameter	Curene®	Placebo
	(N=25)	(N=25)
VAS (in mm)	52.37±6.41	52.79±4.47
WOMAC (Total)	37.88±4.14	37.36±4.08
WOMAC Subscale		
Stiffness	4.52±0.92	4.72±1.02
Pain	8.24±1.56	8.16±1.21
Physical Function	25.12±2.21	24.48±2.71

**Table 4:** Mean change from baseline in total WOMAC.

Visits	Curene® 500mg		Placebo	
	N=25		N=25	
	Mean (SD)	Change from baseline	Mean (SD)	Change from baseline
Baseline (Day 0)	37.88±4.14		37.36±4.08	
Visit 2 (Day 7)	34.88±5.27	3±1.55*∗*∧	36.4±3.88	0.96±1.77
Visit 3 (Day 15)	31.44±4.85	6.44±1.50*∗*∧	34.36±3.79	3±2.22*∗*
Visit 4 (Day 30)	25.84±5.54	12.04±2.89*∗*∧	32.76±3.88	4.6±2.81*∗*
Visit 5 (Day 60)	18.44±4.75	19.44±3.74*∗*∧	30.76±4.84	6.6±3.66*∗*

*∗*Statistically significant (P <0.05) within group; ∧ statistically significant (P <0.05) between group.

Within group analysis by Pair t-test and between group analysis by repeated measures ANCOVA.

**Table 5:** Change from baseline in VAS (in mm).

Visits	Curene® 500mg		Placebo	
	N=25		N=25	
	Mean (SD)	Change from baseline	Mean (SD)	Change from baseline
Baseline (Day 0)	52.37±6.41		52.79±4.47	
Visit 2 (Day 7)	46.59±8.19	5.78±3.62*∗*∧	51.94±4.16	0.86±2.23
Visit 3 (Day 15)	41.71±9.35	10.66±6.08*∗*∧	49.28±4.40	3.51±4.99*∗*
Visit 4 (Day 30)	36.63±8.85	15.74±5.23*∗*∧	46.77±3.84	6.03±4.65*∗*
Visit 5 (Day 60)	27.26±11.95	25.11±8.66*∗*∧	44.83±4.27	7.97±5.29*∗*

*∗*Statistically significant (P <0.05) within group; ∧statistically significant (P <0.05) between group.

Within group analysis by Pair t-test and between group analysis by repeated measures ANCOVA.

**Table 6:** Change from baseline in WOMAC pain score.

Visits	Curene® 500mg		Placebo	
	N=25		N=25	
	Mean (SD)	Change from baseline	Mean (SD)	Change from baseline
Baseline (Day 0)	8.24±1.56		8.16±1.21	
Visit 2 (Day 7)	7.36±1.73	0.88±0.44*∗*∧	8±1.12	0.16±1.03
Visit 3 (Day 15)	6.6±1.66	1.64±0.64*∗*∧	7.64±0.91	0.52±1.16*∗*
Visit 4 (Day 30)	5.8±1.80	2.44±0.72*∗*∧	7.32±1.07	0.84±1.25*∗*
Visit 5 (Day 60)	4.28±1.54	3.96±1.06*∗*∧	6.96±1.43	1.2±1.44*∗*

*∗*Statistically significant (P <0.05) within group; ∧statistically significant (P <0.05) between group.

Within group analysis by Pair t-test and between group analysis by repeated measures ANCOVA.

**Table 7:** Change from baseline in WOMAC stiffness score.

Visits	Curene® 500mg		Placebo	
	N=25		N=25	
	Mean (SD)	Change from baseline	Mean (SD)	Change from baseline
Baseline (Day 0)	4.52±0.92		4.72±1.02	
Visit 2 (Day 7)	4.16±1.07	0.36±0.49*∗*∧	4.64±0.91	0.08±0.57
Visit 3 (Day 15)	3.52±0.96	1±0.58*∗*∧	4.4±0.87	0.32±0.63*∗*
Visit 4 (Day 30)	2.84±1.07	1.68±0.63*∗*∧	4±0.91	0.72±0.61*∗*
Visit 5 (Day 60)	2.12±0.97	2.4±0.82*∗*∧	3.76±1.09	0.96±0.68*∗*

*∗*Statistically significant (P <0.05) within group; ∧statistically significant (P <0.05) between group.

Within group analysis by Pair t-test and between group analysis by repeated measures ANCOVA.

**Table 8:** Change from baseline in WOMAC physical function.

Visits	Curene® 500mg		Placebo	
	N=25		N=25	
	Mean (SD)	Change from baseline	Mean (SD)	Change from baseline
Baseline (Day 0)	25.12±2.21		24.48±2.71	
Visit 2 (Day 7)	23.36±3.05	1.76±1.16*∗*∧	23.76±2.73	0.72±0.98*∗*
Visit 3 (Day 15)	21.32±2.85	3.8±1.22*∗*∧	22.32±2.94	2.16±1.31*∗*
Visit 4 (Day 30)	17.2±3.65	7.92±2.63*∗*∧	21.44±3.03	3.04±1.79*∗*
Visit 5 (Day 60)	12.04±3.12	13.08±2.81*∗*∧	20.04±3.77	4.44±2.63*∗*

*∗*Statistically significant (P <0.05) within group; ∧statistically significant (P <0.05) between group.

Within group analysis by Pair t-test and between group analysis by repeated measures ANCOVA.

**Table 9:** Laboratory parameters (ITT population).

Index	OLNP-08 500 mg			Placebo		
	N=25			N=25		
	Baseline visit	Visit 5	Mean change	Baseline visit	Visit 5	Mean change
Haemoglobin	13.81±1.38	14.67±0.95	0.87±0.91*∗*	13.62±1.02	14.67±0.65	-1.02±0.86*∗*
Platelet count	2.19±0.60	2.99±0.66	0.8±0.53*∗*	2.43±0.66	3.04±0.53	0.61±0.55*∗*
ESR	28.2±4.52	25.12±3.00	3.08±3.12*∗*	27.88±4.01	27.68±3.97	0.2±2.31
RBC	5.04±0.58	5.34±0.54	0.30±0.6*∗*	4.96±0.52	5.41±0.7	0.45±0.63*∗*
WBC	8260±1283.87	8580± 1559.11	320±1360.76	8200±1600.78	8420±1476.76	220±925.11
Random Glucose	142.88±25.67	142.52±18.22	0.36±14.41	141.8±25.22	146.68±18.34	4.88±13.07
S. Creatinine	0.79±0.14	0.79±0.18	0.00±0.17	0.78±0.20	0.92±0.32	0.14±0.30*∗*
BUN	32.88±9.00	36.52±6.37	3.64±8.54*∗*	35.24±8.00	34.36±8.58	0.88±8.1
Bilirubin	0.75±0.22	0.83±0.15	0.08±0.19*∗*	0.72±0.26	0.73±0.24	0.01±0.16
SGOT	31.38±6.38	31.44±5.28	0.06±8.17	29.56±7.88	31.04±5.52	1.48±8.36
SGPT	21.59±8.13	24.56±7.92	2.99±6.5*∗*	21.48±6.25	26.04±5.72	4.56±5.35*∗*
SAP	112.52±24.38	119.76±18.97	7.24±11.4*∗*	116.16±24.47	120.2±20.12	4.04±11.97
S. Sodium	140.16±5.61	138.4±3.57	1.76±5.98	139.5±2.39	137.88±5.92	1.62±5.64
S. Potassium	4.20±0.53	4.28±0.53	0.08±0.66	4.15±0.42	4.57±0.66	0.42±0.67*∗*
S. Albumin	4.07±0.41	4.48±0.76	0.41±0.74*∗*	4.13±0.54	4.57±0.71	0.44±0.72*∗*
Urine PH	6.46±0.66	6.66±0.47	0.2±0.5	6.38±0.39	6.48±0.37	0.1±0.32
Specific gravity	1.01±0.01	1.01±0.00	0.00±0.00	1.01±0.00	1.01±0.00	0.00±0.00

*∗*P value < 0.05, values are presented in absolute value. Within the group analysis by pair t-test.

## Data Availability

The data used to support the findings of this study are available from the corresponding author upon request.

## Abbreviations

AE: Adverse event
ACR: American College of Rheumatology
ANCOVA: Analysis of covariance
BMI: Body mass index
COX-2: Cyclooxygenase-2
LOX: Lipoxygenase
OA: Osteoarthritis
VAS: Visual Analogue Scale
WOMAC: Western Ontario and McMaster Universities Osteoarthritis Index.
